# Long-Term In Vivo Imaging of Multiple Organs at the Single Cell Level

**DOI:** 10.1371/journal.pone.0052087

**Published:** 2013-01-02

**Authors:** Benny J. Chen, Yiqun Jiao, Ping Zhang, Albert Y. Sun, Geoffrey S. Pitt, Divino Deoliveira, Nicholas Drago, Tong Ye, Chen Liu, Nelson J. Chao

**Affiliations:** 1 Departments of Medicine, Duke University Medical Center, Durham, North Carolina, United States of America; 2 Department of Immunology, Duke University Medical Center, Durham, North Carolina, United States of America; 3 Duke Cancer Institute, Duke University Medical Center, Durham, North Carolina, United States of America; 4 Department of Neurobiology, University of Alabama at Birmingham, Birmingham, Alabama, United States of America; 5 Department of Pathology, Immunology and Laboratory Medicine, College of Medicine, University of Florida, Gainesville, Florida, United States of America; Medical University Innsbruck, Austria

## Abstract

Two-photon microscopy has enabled the study of individual cell behavior in live animals. Many organs and tissues cannot be studied, especially longitudinally, because they are located too deep, behind bony structures or too close to the lung and heart. Here we report a novel mouse model that allows long-term single cell imaging of many organs. A wide variety of live tissues were successfully engrafted in the pinna of the mouse ear. Many of these engrafted tissues maintained the normal tissue histology. Using the heart and thymus as models, we further demonstrated that the engrafted tissues functioned as would be expected. Combining two-photon microscopy with fluorescent tracers, we successfully visualized the engrafted tissues at the single cell level in live mice over several months. Four dimensional (three-dimensional (3D) plus time) information of individual cells was obtained from this imaging. This model makes long-term high resolution 4D imaging of multiple organs possible.

## Introduction

The use of microscopy in medicine has revolutionized medical research, diagnosis, and treatment [1]. While microscopes have opened up the world of microbes, cells and tissues, its current use is mostly limited to two dimensional views. The introduction of newer technologies into microscopes, such as confocal and multiphoton excitation laser scanning microscopy, has enabled study of three dimensional structures deep in living tissues [2]. These technologies have allowed imaging of cells and cellular interactions with high resolution in their intact environments. Because there is still a limit as to how deep these microscopic techniques can detect signal in tissue (confocal: 50–100 µm; multiphoton: 300–600 µm) [2], many tissues/organs cannot be well studied due to inaccessibility (e.g., organs in the thoracic cavity). For tissues/organs that have been successfully studied using these novel imaging techniques, surgical exposure is almost always required [3] because even the thickness of dorsal skin exceeds the imaging depth of the multiphoton microscope. Moreover, surgical exposure allows for only limited visualization in time since multiple surgeries would be required in the same area to allow continued visualization over many weeks. This requirement significantly limits the ability to investigate three dimensional structures over time. Moreover, surgery-induced trauma may induce inflammation, potentially interfering with the physiological process [3]. In addition, all of the above-mentioned techniques have only practically been applied to the microscopic study of tissues/organs in small animal models (e.g. mouse models). Currently, there is no viable 3 dimensional microscopic approach to the study of internal human living tissues/organs [3].

Investigators in the field of transplantation have been using an “ear-heart” murine model to study immune tolerance for more than 40 years [4]. In this model, a heart from a newborn mouse is transplanted subcutaneously into the pinna of the ear. All hearts will begin to beat if the surgery is successful. If the heart is accepted (e.g., syngeneic setting), it can survive and beat indefinitely; whereas if the heart is rejected, it will stop beating after about 10 days [5]. We have been using this model for more than 15 years for the study of transplant tolerance in a variety of hematopoietic stem cell transplant settings [5], [6]. We hypothesized that other tissues/organs could also be transplanted into this space in the ear of the mouse and more importantly, the transplanted tissue could function as it would under normal circumstances. Because ear skin is much thinner than dorsal skin, we can image these engrafted tissues using confocal or two-photon microscopes to visualize cellular differentiation and different cellular interactions if these engrafted tissues are able to survive.

In the current study, we demonstrated that a wide variety of adult and fetal live tissues could be engrafted into the pinna of the ear. These engrafted tissues were able to survive for more than 8 weeks. Using the heart and thymus, we further demonstrated that the engrafted tissues function normally as indicated by the heart beating and the donor thymus producing new mature T cells. Finally, we successfully visualized the engrafted tissues at the single cell level in living animals. Since they are externalized but permanent, these tissues can be visualized on a daily basis without the need for sacrificing the animal after each experiment.

## Results

### A Wide Variety of Tissues were Successfully Engrafted in Mouse Ear Pinna and Survived Long-term

**Figure 1:**
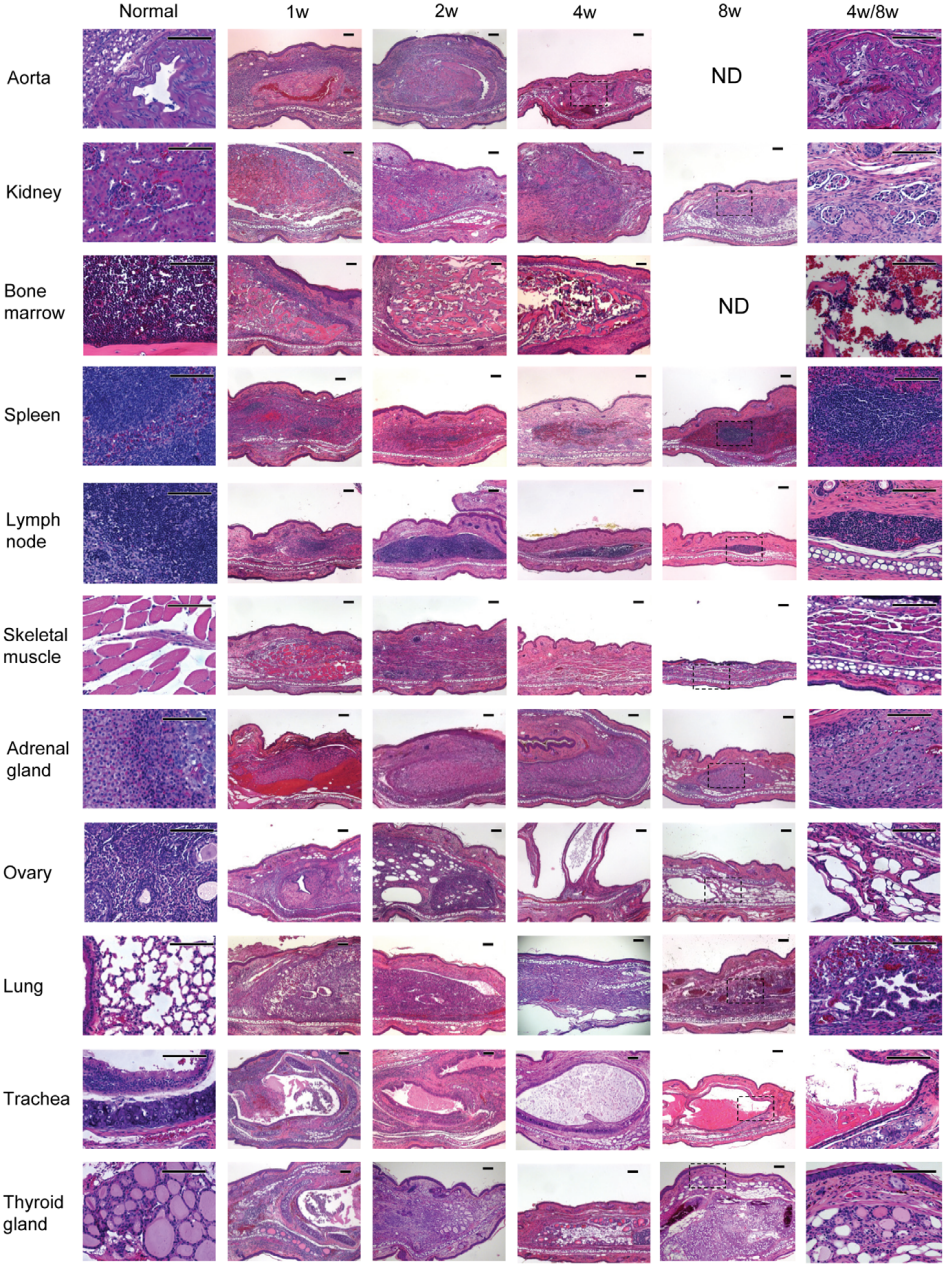
Histological analysis of adult tissues transplanted into ear pinnae (H&E stain, scale bar = 100 µm). B6 CD45.1 adult tissues or pieces of organs were subcutaneously transplanted into syngeneic mouse ear pinnae. Ear-tissues were harvested at different time points post transplantation. Similar experiments have been repeated for at least 3 times.

To test whether different tissues can engraft in mouse ear pinna, we transplanted a variety of tissues and pieces of organs including aorta, kidney, bone marrow, spleen, lymph node, skeletal muscle, adrenal gland, ovary, lung, trachea, and thyroid gland from adult B6 CD45.1 mice into the pinnae of the ear in syngeneic recipients. The recipient mice were sacrificed at different time points after transplantation. The engrafted tissues were then harvested for histological analyses. The results are shown in Figure 1:

- Aorta: it demonstrated a patent vascular structure resembling the “normal aortic tissue.” Since there was no vascular flow, the central lumen was not fully patent at the later date.
- Kidney: renal tissue was observed up to 8 weeks at the implantation site. Glomeruli and interstitial stromal tissue was well preserved.
- Bone marrow: it demonstrated the remodeling of the bony structures with residual cellular matrix at 8 weeks.
- Spleen: lymphoid aggregates were evident in the engrafted splenic tissue.
- Lymph node: the well-preserved lymphoid follicles were observed at the implantation site for up to 4 weeks.
- Skeletal muscle: viable skeletal muscle exemplified by the presence of the striation of the muscle fibers and the peripheral localization of the nuclei were demonstrated.
- Adrenal gland: it showed the adrenal cortical cells with clear cytoplasm. The cells were forming sheets resembling the “normal” adrenal tissue.
- Ovary: the ovary sections showed normal ovarian tissue. The germ cells appeared to be degenerated quickly.
- Lung: the bronchial and alveolar structures were well preserved up to 8 weeks in the engrafted lung tissue.
- Trachea: the tracheal tissue was exemplified by the presence of cartilage and the lining epithelium. Well preserved cilia were observed.
- Thyroid gland: the follicular structure that is characteristic for thyroid tissue could be observed in the engrafted thyroid gland up to 4 weeks after implantation.

**Figure 2:**
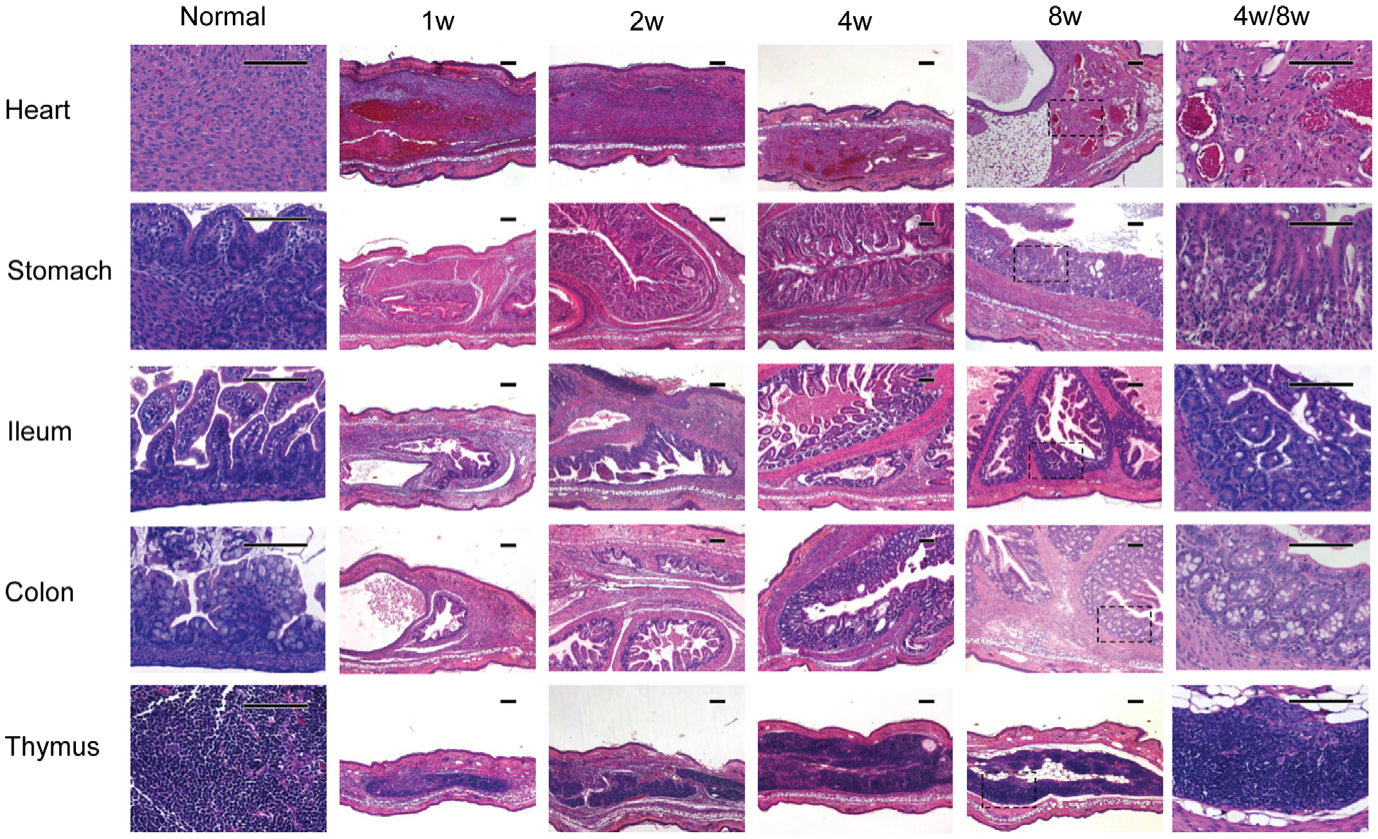
Histological analysis of fetal tissues transplanted into ear pinnae (H&E stain, scale bar = 100 µm). B6 CD45.1 near-term fetal tissues were subcutaneously transplanted into syngeneic mouse ear pinnae. Ear-tissues were harvested at different time points post transplantation. Similar experiments have been repeated for at least 3 times.

We also used near-term fetal donors because some of the tissues could not be obtained in a sterile manner from adult donors (e.g., intestine) or the adult tissues did not function as well as fetal tissues (e.g., thymus). The histological results are shown in Figure 2:

- Heart: cardiac muscle could be observed up to 8 weeks after transplantation.
- Stomach: the gastric tissue could be well maintained up to 8 weeks. The parietal cells and the chief cells were noticed in the glandular compartment.
- Ileum: the properly oriented small intestinal villi were evident in the engrafted ileum. At the base of the crypts, Paneth cells were readily seen. The intestinal lumen was also patent.
- Colon: a patent colonic structure was seen in the engrafted colon. The colonic mucosa was well-preserved. The lining epithelium contained normal mucin.
- Thymus: the lymphoid tissue was seen at the implantation site, which resembled the “normal” thymic tissue.

These histological data demonstrated that a wide variety of tissues survived in this area of the ear for up to 8 weeks. Histologically the morphology of these tissues was preserved.

### Survival and Function of Neonatal Heart Tissue Engrafted into Ear Pinna

**Figure 3:**
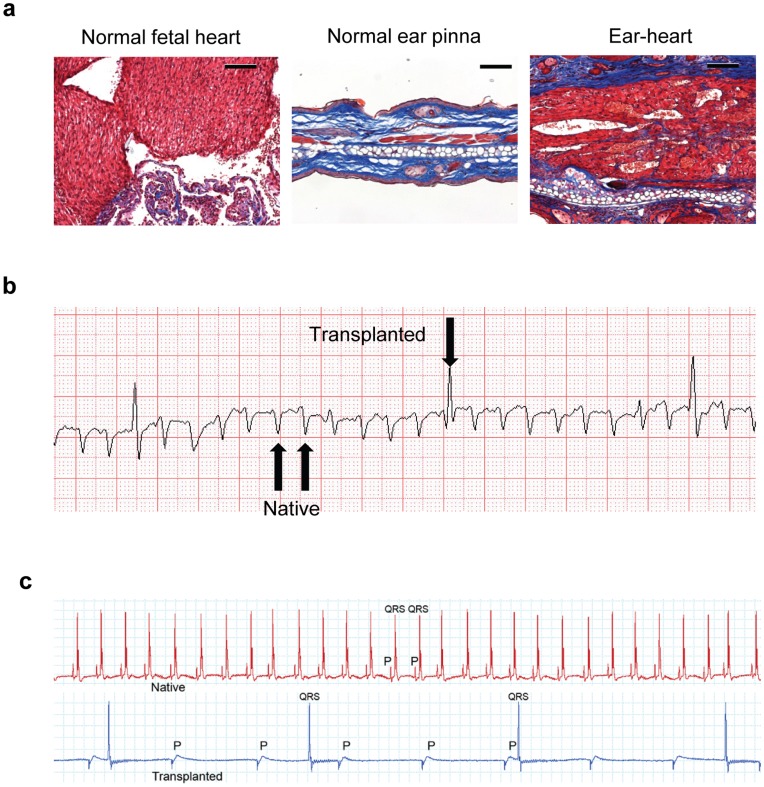
Survival and function of near-term fetal heart tissue engrafted into ear pinna. B6 CD45.1 near-term fetal heart tissue was transplanted subcutaneously into syngeneic mouse ear pinnae. ECG was performed 7 weeks after transplantation. Ear-tissues were harvested from a different mouse 8 weeks post transplantation. (a) Masson’s Trichrome staining of ear-heart tissue (Scale bar = 100 µm). Muscle fibers and cytoplasm are stained in red, collagen and mucin are stained in blue; (b) ECG on a mouse engrafted with a near-term fetal ear-heart. Note the transplanted heart QRS complex is larger in amplitude given the placement of the unipolar ECG electrode in the pinna of the ear. (c) Simultaneous local ECG recordings of native and transplanted hearts. Similar experiments have been repeated for at least three times.

One of the critical questions is whether the engrafted tissues can function. To answer this, we first verified whether cardiac muscle truly survived in the transplanted heart graft using Masson’s Trichrome stain. As shown in Figure 3a, the transplanted heart muscle as shown in red could be easily visualized in the space anterior to the cartilage of the ear. As expected, the transplanted fetal heart continued to beat more than 100 days after transplantation (Video S1). Moreover, an electrocardiogram (ECG) that was performed more than 60 days after transplantation demonstrated that the ectopic heart could produce an expected ventricular depolarization (Figure 3b). Local bipolar recordings of another transplanted heart also demonstrated intact atrial and ventricular depolarization with intermittent AV nodal conduction (Figure 3c).

### T Cell Reconstitution in Mice Engrafted with Thymic Tissue in the Pinna of the Ear

**Figure 4:**
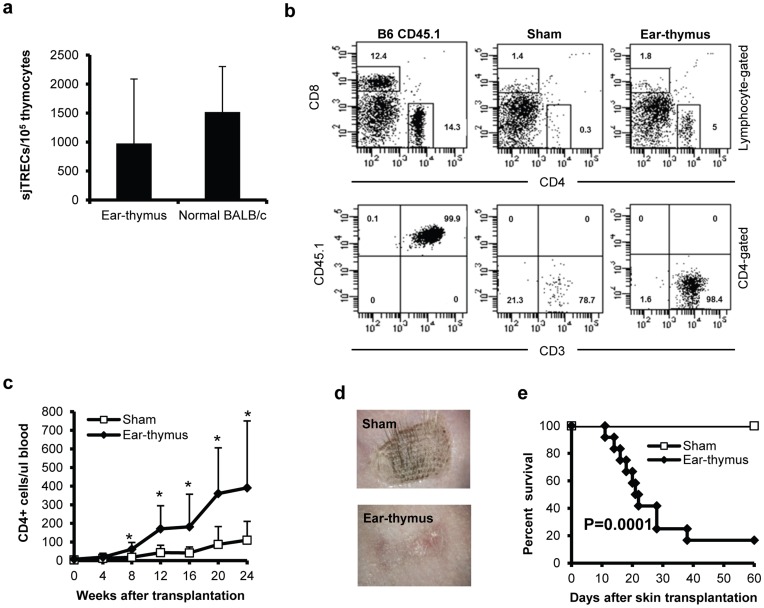
T cell reconstitution in mice engrafted with ear-thymus. Thymuses from B6 CD45.1 neonatal mice (<48 hours old) were transplanted into the ear pinnae of BALB/c nude mice. T cell reconstitution was monitored phenotypically and functionally. (a) Thymic grafts were harvested 5 months after transplantation. siTREC numbers were determined in thymocytes. This is a representative of three similar experiments. Each group contained 3 animals. P = not significant. (b&c) Phenotypic T cell recovery. Peripheral T cell recovery was monitored in peripheral blood by flow cytometry. This is a representative of two similar assays with similar results. Each group contained more than 16 animals. ^*^P<0.05; (d&e) Skin transplantation. More than three months after ear-thymic transplantation, ear-thymus recipients were transplanted with third-party C3H/HeJ skin grafts. Survival of skin grafts was monitored daily after transplantation. Pictures (d) were taken 5 weeks after skin grafting. The combined results from two similar experiments are shown. Each group contained 10–12 animals.

To further demonstrate that it is not simply the survival of the organ in this space but that the organ can actually function as would be predicted based on its origin, we transplanted a thymus from a B6 CD45.1 neonatal mouse (<48 hours old) into the pinna of a BALB/c nude animal (CD45.2). We then monitored the development of T cells. Since nude animals have competent lymphoid precursors but lack a thymus, new T cells, especially functional T cells would have to be generated from the transplanted thymus. Indeed, we were able to detect signal joint T-cell receptor rearrangement excision circles (siTREC, a marker for active thymopoiesis [7]–[9]) in nude mice engrafted with thymic tissue in the pinna of the ear (Figure 4a). Moreover, since the T cells from the host and within the thymus differ in the CD45 isoform, we were able to determine the origin of the peripheral T cells as shown in Figure 4b (thymus donor: CD45.1^+^, host: CD45.1^−^). New T cells, especially CD4^+^ cells, were readily detectable in the peripheral blood beginning approximately 8 weeks after transplantation of the donor thymus (Figure 4b, c). Moreover, these T cells arose predominantly from the BALB/c marrow precursors, which were then educated in the donor thymus (Figure 4b). As demonstrated in Figure 4c, ear-thymus recipients had significantly higher numbers of CD4^+^ T cells in peripheral blood than the sham transplanted group 8 weeks post transplantation (P<0.05).

To determine whether transplantation of thymic tissues in the ear pinna truly restores T cell function in nude mice, we transplanted third-party skin grafts from C3H/HeJ into the animals that had received either a piece of thymic tissue or just a sham operation. As can be seen in Figure 4d, e, the animals receiving the thymic transplant were able to reject the donor graft within 60 days after skin transplantation while the animals receiving a sham thymic transplant did not reject their skin graft (P<0.0001).

### Ear-tissue can be Visualized at the Cellular Level in Living Animal

**Figure 5:**
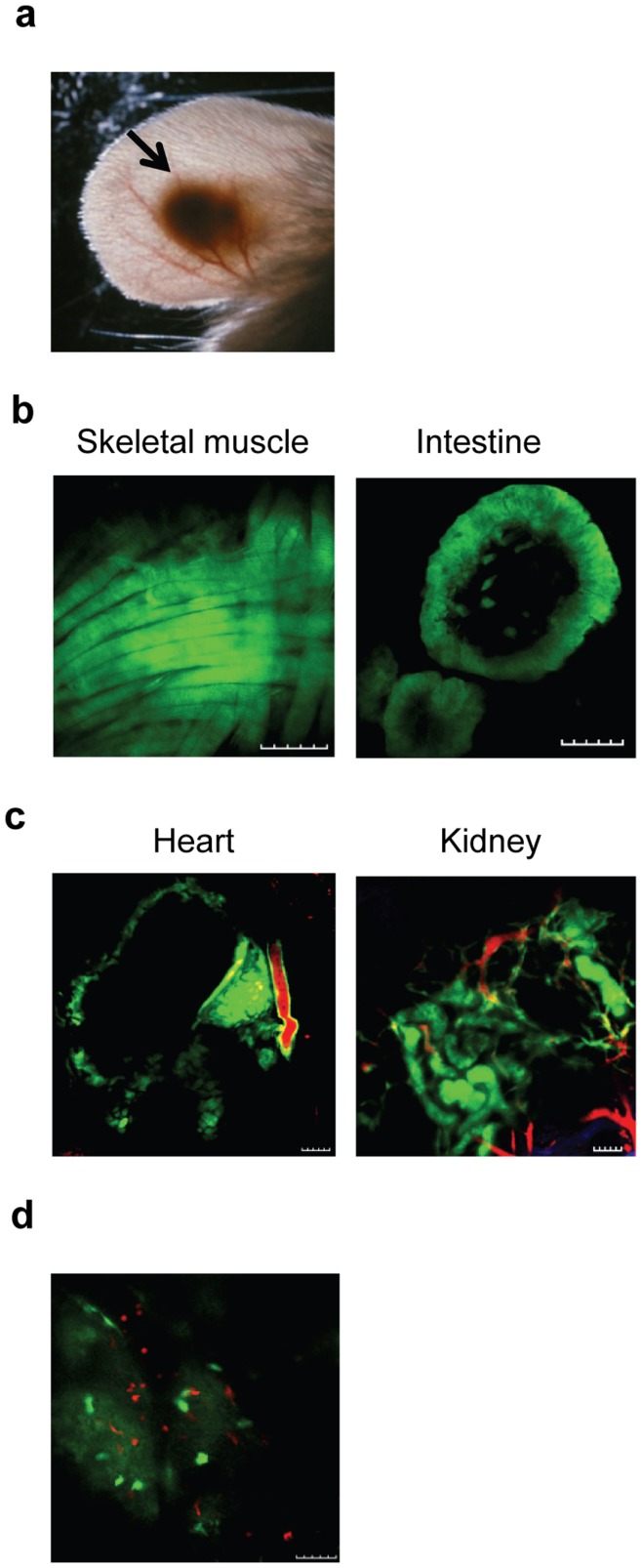
Ear-tissue can be visualized at the cellular level in living animal. (a) This picture showing the location of an engrafted heart graft in the ear pinna. (b) Skeletal muscle and small intestine tissues from EGFP mice were subcutaneously transplanted into the ear pinnae of BALB/c nude mice. The images were obtained 9 (muscle) and 22 (intestine) weeks after transplantation. The images were taken 107 µm (muscle) deep from surface. The depth for intestine could not be determined. (c) Heart and kidney tissues from EGFP mice were subcutaneously transplanted into the ear pinnae of BALB/c nude mice. Rhodomin B conjugated dextran was injected i.v. to visualize the blood vessels. The images were obtained 9 (heart) and 2 (kidney) weeks after transplantation. Green = EGFP; red = dextran. (d) EGFP^+^ neonatal thymic tissue was subcutaneously transplanted into the ear pinnae of BALB/c nude mice. Eight weeks later, the mice were irradiated and transplanted with DsRed^+^ T cell depleted bone marrow cells. The image was taken 4 weeks after bone marrow transplantation at the depth of 103 µm. Green = EGFP; red = DsRed. Scale bar = 100 µm.

The data above clearly demonstrated that not only were the tissues surviving in this resident space but also that they were able to function as would be expected. To demonstrate whether the engrafted tissues can be imaged in the ear pinna, we utilized enhanced green fluorescent protein (EGFP)- or discosoma red fluorescent protein (DsRed)-transgeneic donor mice. More than two weeks after transplantation, engrafted ear-tissues were imaged in live animals using two-photon microscopy. Figure 5a shows the location of the transplanted organs in the ear pinna. Images of engrafted fluorescent tissues are shown in Figure 5b, c, d. Both EGFP^+^ muscle cells and small intestinal villi engrafted in the ear pinnae could be imaged clearly (Figure 5b). As shown in Figure 5c and Video S2, EGFP^+^ heart muscle fiber was clearly evident in the beating heart graft. EGFP^+^ glomeruli could also be recognized in the ear-kidney graft (Figure 5c and Video S3). In Figure 5c and Videos S2 and S3, rhodamin B labeled dextron (shown in red) was injected to visualize the blood vessel. In order to study cell-to-cell interaction using this model, we transplanted EGFP^+^ ear-thymus recipients with DsRed^+^ T cell depleted bone marrow cells 8 weeks after thymic transplantation. Imaging was then performed 4 weeks after bone marrow transplantation. As shown in Figure 5d and Video S4, single EGFP^+^ and DsRed^+^ cells could be clearly recognized as deep as 143 µm below the surface. These data clearly demonstrated that tissues engrafted in the pinna of the ear could be visualized at the single cell level in living animals.

### Visualizing Radiation-induced Thymocyte Apoptosis in Ear-thymus Graft

**Figure 6:**
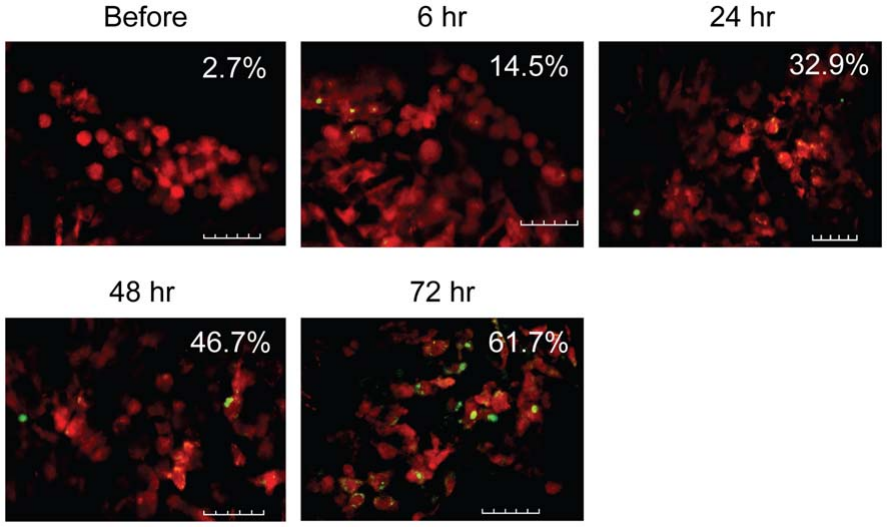
Visualizing radiation-induced thymocyte apoptosis in ear-thymus graft. A nude BALB/c chimera containing DsRed^+^ hematopoietic cells were transplanted with a thymus from a B6 CD45.1 neonatal mouse (<48 hours old) into the ear pinna. Five weeks later, the ear pinna containing thymus graft was irradiated with 8.5 Gy. Cell apoptosis was then followed over time by in vivo two-photon imaging after injection of FAM-FLIVO. The images were taken 113 to 137 µm deep from surface. Representative pictures from each group are shown. The percentages of apoptotic cells are shown. Similar experiments have been repeated for three times. Green = EGFP; red = apoptotic cells. Scale bar = 50 µm.

To demonstrate whether this novel model can be used to study biological questions, we followed the fate of thymocytes in the ear-thymus graft after irradiation using intravital two-photon imaging. A nude BALB/c chimera containing DsRed^+^ hematopoietic cells were transplanted with a thymus from a B6 CD45.1 neonatal mouse (<48 hours old) into the ear pinna. Five weeks later, the ear pinna containing thymus graft was irradiated with a lethal dose of radiation (8.5 Gy). Cell apoptosis was then followed in the thymus graft over time by in vivo two-photon imaging after injection of carboxyfluorescein (FAM)-FLIVO™, a green fluorescent probe specific for apoptosis. In this model, almost all hematopoietic cells including thymocytes are DsRed^+^ (red) and the apoptotic cells are green. As demonstrated in Figure 6, only 2.7% apoptotic cells were observed before irradiation. Apoptotic cells were increased to 14.5% of total thymocytes as early as 6 hours after irradiation. The numbers of apoptotic cells kept increasing over time. By 72 hours, 61.7% of thymocytes were apoptotic.

### Visualizing Hematopoietic Cell Engraftment in Spleen Engrafted in the Mouse Pinna

**Figure 7:**
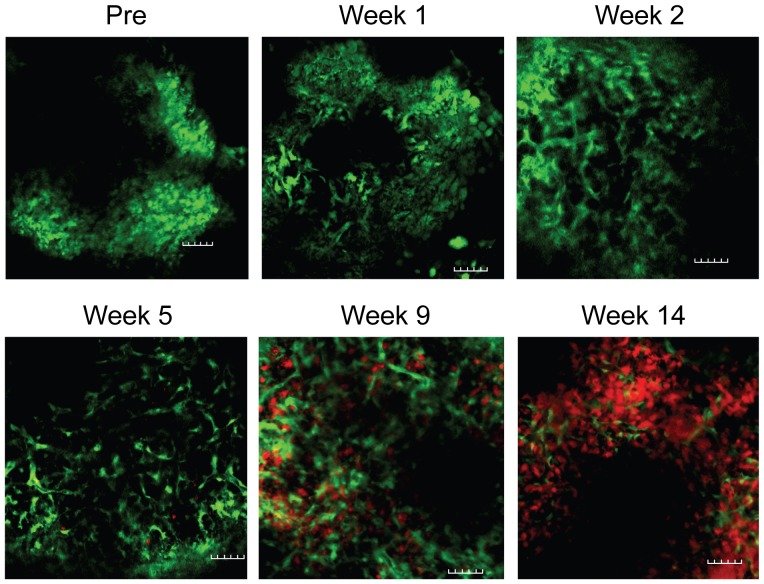
Visualizing hematopoietic cell engraftment in spleen engrafted in the mouse pinna. A BALB/c nude recipient of EGFP^+^ spleen was lethally irradiated and then transplanted with DsRed^+^ TCD BM cells. Engraftment of hematopoietic cells (red) in ear-spleen (green) was tracked over time by two-photon microscopy. The images were taken 94–169 µm deep from surface. Similar experiments have been repeated twice. Green = EGFP; red = DsRed. Scale bar = 50 µm.

To further demonstrate that this model is suitable for studying biological questions using in vivo imaging, we tracked hematopoietic cell engraftment in spleen engrafted in the mouse pinna. A BALB/c nude recipient of EGFP^+^ spleen was lethally irradiated and then transplanted with DsRed^+^ T-cell-depleted bone marrow (TCD BM) cells. Engraftment of hematopoietic cells (red) in ear-spleen (green) was tracked over time by two-photon microscopy. As shown in Figure 7, EGFP^+^ lymphocytes were evident in ear-spleen graft before irradiation and transplantation. One week after irradiation and stem cell transplantation, some of the host EGFP^+^ lymphocytes had disappeared. By two weeks, the majority of the host EGFP^+^ lymphocytes had disappeared and non-hematopoietic cells survived. DsRed^+^ donor cells started to appear in the spleen graft starting from 5 weeks after stem cell transplantation. The numbers of DsRed^+^ donor cells kept increasing in the following weeks. By week 9, a large amount of DsRed^+^ donor cells were evident in the ear-spleen graft. By week 14, almost all lymphocytes found in the spleen graft were from the DsRed^+^ donor.

## Discussion

Our data have demonstrated that a wide variety of adult and fetal tissues can be successfully engrafted in the pinnae of mouse ear (Figure 1, 2). Using two different models (Figure 3, 4), we have further demonstrated that the transplanted tissues are functional. Based on these observations, we conclude that these transplanted organs can function in a physiological condition. Because skin on the mouse ear pinna is extremely thin (Figure 1, 2, 3), this model allows in vivo high resolution 4 dimensional imaging of multiple tissues in real time. The imaging data obtained through this transplantation model can be used to understand basic physiological questions of many of these organs.

The ability to image cellular events in vivo will allow for direct visualization of complex biological and pathophysiological processes at the cellular or even subcellular level. This approach is analogous to performing histological sectioning in vivo without having to sacrifice the animal and thereby allowing continuous visualization over time. The immediate use of this model is to study organs in the thoracic cavity such as thymus, heart, and lung. Currently, it is almost impossible to image these organs because of the surgical difficulty and vibration induced by breathing and the beating heart. We have already demonstrated that we can image individual cells in transplanted heart (Figure 5c and Video S2) and thymic tissues (Figure 5d and Video S4). Unlike most of the currently existing models that required surgical exposure [3], high resolution in vivo imaging using this model can be performed repeatedly over sequential time points spanning several months. This may allow us to trace a specific cell over a long period of time so that we can understand the physiological processes at the cellular level. For example, thymopoiesis has already been successfully studied at the single cell level using two-photon imaging of thymic tissues ex vivo [10]–[12]. Because thymocytes can be imaged at the cellular level over time using our ear-thymus model (Figure 5d and 6), this model can be used to study different stages of thymopoiesis such as positive and negative selection at the cellular level. In addition, this model can also be used to study hematopoietic cell engraftment (Figure 7).

Since multiple immunodeficient mouse strains are available [13], [14], we postulate that human tissues/organs can also be transplanted into the mouse ear pinna especially in the NOD.Cg-Prkdc^scid^ (NOG) mouse. The applicability of this model to human tissues will open the door for the study of human physiology and pathophysiology in intact live tissues/organs directly.

There is a potential limitation of this model in that the physiological process in the engrafted tissues may not be exactly the same as that in naïve tissues. This is especially true for blood vessels, lung, intestine, and stomach where blood, air or gastric contents are important for normal function. Nevertheless, the ability to visualize homeostatic and pathologic processes in real time using the model reported here adds a vital extra dimension to our understanding of cell-cell interaction and cellular function.

## Methods

### Ethics Statement

All experiments were performed under research protocols approved by the Duke University Animal Care and Use Committee and were in accordance with the National Institutes of Health Guide for the Care and Use of Laboratory Animals.

### Mice

C3H/HeJ mice (H2^k^) and B6.Cg-Tg(CAG-DsRed*MST)1Nagy/J mice (H2^b^, termed “DsRed”) were purchased from The Jackson Laboratories (Bar Harbor, ME). C57BL/Ka, CD45.1, Thy1.1 (H2^b^, termed“B6 CD45.1”) and EGFP^+^ C57BL/Ka, CD45.2, Thy1.1 (H2^b^, termed “EGFP”) were originally generated by Dr. Irving Weissman (Stanford University, Palo Alto, CA). The breeders of both B6 CD45.1 and EGFP mice were kindly provided by Dr. Jos Domen (Duke University, Durham, NC). Athymic BALB/c nude mice (nu/nu, H2^d^) were obtained from the Cancer Center Isolation Facility at Duke University. Animals were housed in sterile microisolator cages in a specific pathogen-free facility throughout the study.

### Surgery

The ear-tissue transplantation was performed according to a published method from our laboratory [5]. Briefly, the dorsum of the pinna of an adult mouse was cleaned and a pouch about 5 mm in diameter was prepared. Tissues or pieces of organs were placed in the pouch. Residual fluid was cleared from the pouch with a cotton swab after transplantation. The skin transplantation procedure was based on a protocol published by Markees et al [15]. Briefly, tail skin was removed from sacrificed donors and cut into 0.5×0.5 cm^2^ pieces. The dorsal surface of anesthetized recipient mice was shaved and washed with iodine solution, and rinsed with an alcohol swab. A graft bed was prepared with fine scissors by removing an area of epidermis and dermis down to the level of the intrinsic muscle. Grafts were fitted to the prepared bed, sutured with 6-0 surgical suture, and then covered with an adhesive plastic bandage. After 7 days, the bandage and suture were removed. Skin graft survival was assessed everyday by visual and tactile examination. Rejection was defined as the first day when the entire epidermal surface of the graft was less than 10%.

### Histology

Ear-tissues were fixed in 10% formalin, embedded in paraffin and cut into 5-µm-thick sections. The sections were stained with hematoxylin-eosin (H&E) or Masson’s Trichrome stain for morphological evaluation. Images were acquired with an AxioCam MRc digital camera mounted on an Axiovert 200 inverted microscope (Carl Zeiss Microimaging, Thornwood, NY). A-Plan 10x/0.25 and LD-Plan NEOFLUAR 20X/0.4 and 40X/0.6 objectives were used. Images were recorded using AxioVision Rel. 4.5 software (Carl Zeiss Microimaging). The original, unmodified pictures were used.

### Flow Cytometry

Fifty µl of heparinized peripheral blood was stained with monoclonal antibodies for 15 minutes at room temperature. Red cells in the stained whole blood samples were then lysed by FACS lysing solution (Becton Dickinson, San Jose, CA). Fifty µl of Flow-Count fluorospheres (Beckmen Coulter) was added before flow cytometric analysis. The stained cells were analyzed using a FACSCanto flow cytometer (BD) equipped with FACSDiva software. The absolute counts were calculated using the following formula: Absolute count (cells/µl blood) = (Total number of cells counted/Total number of fluorospheres counted) × Flow-Count fluorosphere concentration.

### ECG Recording

Mice were anesthetized using 250 mg/kg avertin following institutional guidelines. Anesthetized mice were placed on a heating pad (37°C) for temperature control. ECG recordings were obtained with subcutaneously placed 29-gauge needle electrodes connected to a ML138 Octal Bioamp (ADInstruments Colorado Springs, CO) and a Powerlab 16/30 acquisition system (ADInstruments) in both forelimbs and hindlimbs to create Wilson’s Central Terminal. One or two leads were placed in the pinna of the ear to allow for unipolar or local bipolar recordings respectively. ECGs were sampled at 2 kHz, filtered between 0.3 Hz and 500 Hz and analyzed with LabChart Pro 7.2 software (ADInstruments).

### Mouse sjTREC Assay

Molecules of sjTRECs were quantitated by real-time polymerase chain reaction as previously described [8]–[9].

### In vivo Imaging

In vivo imaging of ear-tissues was performed using a two-photon microscope (FluoView FV1000, Olympus, Central Valley, PA). This microscope was equipped with a 680–1050 nm tunable ultrafast laser (Spetra-Physics, Mountain View, CA). The mice were anesthetized with isoflurane (Butler Animal Health Supply, Dublin, OH) and the ear pinna was fixed to the stage using double-edged tape. EGFP and FAM-FLIVO™ (ImmunoChemistry Technologies, Bloomington, MN) was excited by 860–980 nm laser and detected using a 495–540 nm bandpass filter. DsRed was excited by 980 nm laser and detected using a 575–630 nm bandpass filter. Collagen was imaged by second harmonic generation using 860–920 nm excitation and 420–460 nm detection. Rhodomin B conjugated dextran (70,000 MW, Molecular Probes, Inc, Eugene, OR) was excited by 860 nm laser and detected using a 575–630 nm bandpass filter. Imaging was performed 10 minutes to 2 hours after injection of dextran (i.v., 0.5 mg/mouse). The images and movies were made using FluoView (Olympus) or Imaris (Bitplane, Zurich, Switzerland) software.

### Hematopoietic Stem Cell Transplantation

This procedure was performed according to a previously publication from our laboratory [16]. Briefly, BALB/c nude mice were first irradiated with a lethal dose of radiation (8.5 Gy). The mice were then infused with 1×10^7^ DsRed^+^ T-cell-depleted bone marrow cells intravenously.

### In vivo Apoptosis Assay

This assay was performed according to the manufacturer’s protocol. Mice were injected i.v. with 8 µg of FAM-FLIVO™ (ImmunoChemistry Technologies, Bloomington, MN). Sixty minutes later, mice were imaged by intravital two-photon microscopy. The percentages of positive cells were calculated by the use of Imaris software (Bitplane).

## Supporting Information

Video S1 **B6 CD45.1 near-term fetal heart tissue was transplanted subcutaneously into syngeneic mouse ear pinnae.** Movie was filmed 6 months after transplantation. This is a representative of 6 similar experiments.
(AVI)

Video S2 **Heart and kidney tissues from EGFP mice were subcutaneously transplanted into the ear pinnae of BALB/c nude mice.** Rhodomin B conjugated dextran was injected i.v. to visualize the blood vessels. The Z stacks were obtained using two-photon microscope 9 weeks after transplantation. Green = EGFP; red = dextran. These are the representative movies of at least 5 experiments.
(MOV)

Video S3 **Heart and kidney tissues from EGFP mice were subcutaneously transplanted into the ear pinnae of BALB/c nude mice.** Rhodomin B conjugated dextran was injected i.v. to visualize the blood vessels. The Z stacks were obtained using two-photon microscope 9 weeks after transplantation. Green = EGFP; red = dextran. These are the representative movies of at least 5 experiments.
(AVI)

Video S4 **EGFP^+^ neonatal thymic tissue was subcutaneously transplanted into the ear pinnae of BALB/c nude mice.** Eight weeks later, the mice were transplanted with DsRed^+^ T-cell-depleted bone marrow cells. The image was taken 4 weeks after bone marrow transplantation starting at the depth of 93 µm from the surface. Green = EGFP; red = DsRed.
(AVI)
